# Case report: Isolated axillary lymph node metastasis in high-risk endometrial cancer

**DOI:** 10.3389/fonc.2023.1271821

**Published:** 2024-03-18

**Authors:** Yi-Ju Amy Chen, Myoe Oo, Yiqing Xu

**Affiliations:** ^1^ Division of Gynecologic Oncology, SUNY Downstate Health Sciences University, Brooklyn, NY, United States; ^2^ Department of Internal Medicine, Maimonides Medical Center, Brooklyn, NY, United States; ^3^ Department of Hematologic Oncology, Maimonides Medical Center, Brooklyn, NY, United States

**Keywords:** endometrial cancer, axillary lymph node, isolated metastasis, systemic treatment, radiotherapy

## Abstract

**Introduction:**

There are risks of developing distant metastases over time for both early- and advanced-stage endometrial cancer. Axillary lymph node metastasis as the first site of recurrence, whether isolated or non-isolated, is uncommon, and there are currently no established treatment guidelines for such cases. This study highlights four cases of recurrent endometrial cancer that manifested axillary lymph node metastasis, providing a comprehensive review of their distinctive clinical behavior and the treatment strategies employed.

**Methods:**

We reviewed and compared four cases of recurrent endometrial cancer that developed axillary lymph node metastasis following adjuvant treatment. Patients’ perspectives were also discussed.

**Results:**

All four patients had aggressive endometrial histology, including high-grade serous carcinoma and carcinosarcoma. The stages at presentation were stages I and III, with laparotomy or laparoscopy used as the initial surgical approach. Axillary lymph node metastasis was the primary site of recurrence in three cases. Of the three patients with isolated axillary lymph node metastasis, two had long-term survival after aggressive locoregional treatment comprising surgery and radiation.

**Conclusion:**

Axillary lymph node metastasis as the first site of recurrence is rare, even in high-risk endometrial cancer. In addition to systemic chemotherapy, aggressive locoregional treatment can potentially maximize the chance of long-term disease control.

## 1 Introduction

Endometrial cancer is the most common gynecological cancer in the United States, with an estimated 66,200 new cases diagnosed by 2023 (1). The 5-year relative survival rate is 94.9% for localized uterine cancer and decreases significantly to 69.8% and 18.4% in regional and distant stages, respectively (1). Approximately 6%–10% of early-stage endometrial cancers (2) and 27% of advanced-stage endometrial cancers develop distant metastasis over time (3, 4). The most common sites of distant metastasis include the pelvic and para-aortic lymph nodes, vagina, peritoneum, and lungs (5). High-risk histologies, such as serous carcinoma and carcinosarcoma, can spread hematogenously to the parenchyma of the liver, spleen, supraclavicular lymph nodes, and even the brain (6). Axillary lymph node metastasis presenting as the first site of recurrence, whether isolated or non-isolated, is uncommon and has not been widely reported. We present four cases of recurrent endometrial cancer that developed axillary lymph node metastasis after completing adjuvant treatment and provide an in-depth discussion of their distinctive clinical behavior and the various treatment strategies employed.

## 2 Case description

### 2.1 Case 1

**Figure 1:**
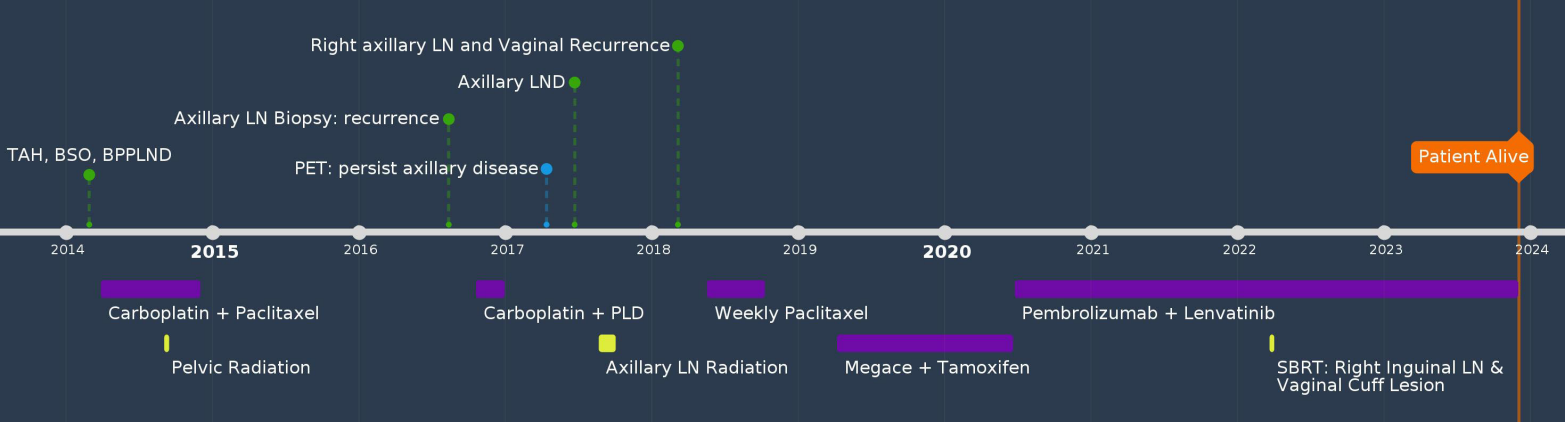
Timeline of Case #1.

A 60-year-old female G0P0, with no significant past medical or family history, had atypical glandular cells on routine Pap smear and endometrioid adenocarcinoma on dilation and curettage. She underwent total abdominal hysterectomy (TAH), bilateral salpingo-oophorectomy (BSO), bilateral pelvic and para-aortic lymphadenectomy (LND), and pelvic washing in 2014. Pathology showed uterine serous carcinoma (two of 50 mm myometrial invasion and pT1a) with one pelvic lymph node involvement (pN1), FIGO stage IIIC1. The peritoneal fluid cytology was positive for malignant cells. She received adjuvant treatment with three cycles of carboplatin and paclitaxel, followed by pelvic radiation and an additional three cycles of chemotherapy. Twenty-nine months after her initial diagnosis, she palpated an enlarged left axillary lymph node was confirmed to be a metastatic high-grade papillary adenocarcinoma by biopsy. PET/CT showed mildly increased FDG uptake in the left axillary lymph nodes that measured 1.1 cm, there was no hypermetabolic adenopathy or other sites of metastasis. Since the patient had residual neuropathy after the first-line treatment and strongly preferred a treatment that caused minimal alopecia, she was treated with four cycles of carboplatin and liposomal doxorubicin. A repeat workup showed stable disease without distant metastasis. She underwent left axillary LND which revealed five of 19 lymph nodes were positive for metastatic serous carcinoma. The patient then received radiation 5040cGy to the left axillary area. Five months after radiation, a physical examination revealed a palpable enlarged inguinal lesion and a palpable vaginal mass. Biopsy confirmed both as recurrent uterine serous carcinoma. Immunohistochemistry (IHC) for mismatch repair (MMR) protein was not performed at the time of the initial diagnosis. However, next generation sequencing (NGS) performed at the time of vaginal recurrence showed the tumor to be microsatellite stable, with a tumor mutational burden of 4 mut/Mb, and p53 mutation. She received six cycles of weekly paclitaxel over a five-month period. She was then switched to alternating megestrol acetate and tamoxifen after CT revealed mild progression, and the disease remained stable for 14 months. The treatment was later changed to pembrolizumab and lenvatinib. She also received stereotactic body radiation therapy to the vaginal and right inguinal lesions while continuing on pembrolizumab and lenvatinib to this date for a total of 37 months, and cell-free DNA test for minimal disease showed no detectable tumor DNA. She is currently alive and doing well 117 months after the initial diagnosis (**Figure 1**).

Patient perspectives: The patient was very optimistic about her prognosis. She was glad that she followed our recommendations for locoregional treatments. She wanted to balance her quality of life with longevity of life and treatment with curative intent. The side effects of the current treatment were greatly diminished after we extended the treatment interval of pembrolizumab to every 6 weeks and gave her a week’s break every 4 weeks with lenvatinib.

### 2.2 Case 2

**Figure 2:**
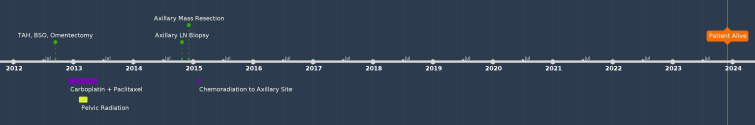
Timeline of Case #2.

A 70-year-old female (G2P2), with hypertension, presented with postmenopausal bleeding (PMB). Her aunt had breast cancer. She was diagnosed with high-grade uterine carcinosarcoma after an endometrial biopsy in 2012. The patient underwent TAH, BSO, omentectomy, and pelvic washing. The pelvic lymph nodes were not accessible because of extensive adhesions at the time of surgery. Final pathology showed uterine carcinosarcoma, pT1a, with one of 22 mm myometrial invasion and pathological stage IA. Peritoneal fluid cytology results were negative. NGS and MMR analyses were not performed. She was treated with six cycles of adjuvant carboplatin and paclitaxel, followed by pelvic external beam radiation and vaginal cuff brachytherapy. Twenty-five months after the initial diagnosis, the patient had an enlarged right axillary lymph node, and biopsy showed metastatic carcinosarcoma. PET/CT showed no other sites of recurrence. She underwent right axillary LND, and pathology confirmed metastatic uterine carcinosarcoma in one out of eight lymph nodes. The patient declined systemic therapy at the time of recurrence but agreed to undergo chemoradiation. She received concurrent chemoradiation at the axillary site at 4,500 cGy and three doses of weekly cisplatin as a radiosensitizer. Since then, she has been under surveillance and remains cancer-free. She is currently alive and doing well, 134 months after the initial diagnosis (**Figure 2**).

Patient perspectives: The patient was grateful for her response to the previous aggressive locoregional treatment of recurrent axillary disease, which led to a favorable outcome. She was motivated to remain vigilant and undergo regular surveillance examinations and scans to detect recurrence as early as possible.

### 2.3 Case 3

**Figure 3:**
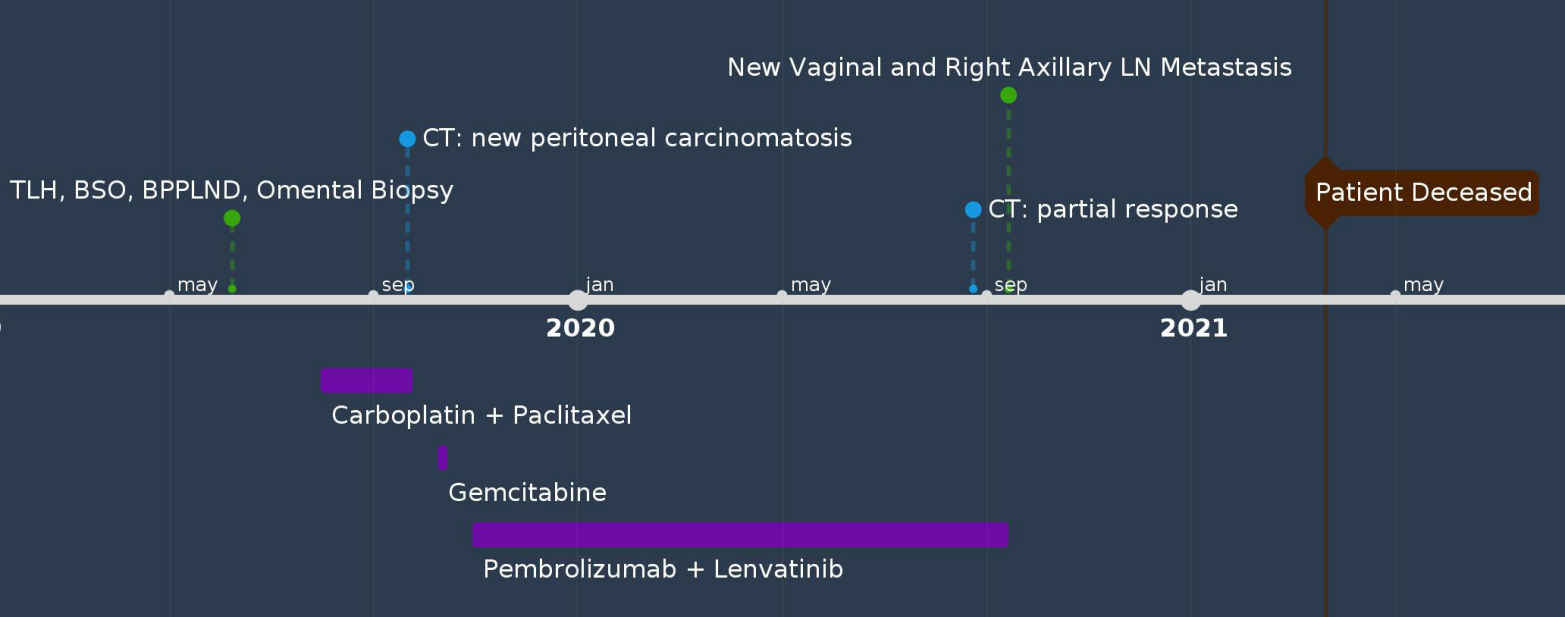
Timeline of Case #3.

A 68-year-old female (G10P5) with a history of Parkinson’s disease, diabetes, and hyperlipidemia presented with PMB and abdominal pain. The patient had no family history of cancer. Endometrial biopsy revealed carcinosarcoma. She underwent robot-assisted total laparoscopic hysterectomy (RATLH), BSO, bilateral pelvic and para-aortic LND, and omental biopsy in 2019. The final pathology showed carcinosarcoma of the uterus (involving 90% of the myometrium, 20 of 22 mm). There was pelvic (11 of 20 positive) and para-aortic lymph node (five out of seven positive) involvement with extranodal spread in para-aortic lymph nodes (FIGO stage IIIC2). The carcinoma component is a mixture of serous and dedifferentiated carcinomas. NGS showed that the tumor was microsatellite stable, with a tumor mutational burden of 8 mut/Mb, BRD4 mutation, and p53 mutation. Shortly after two cycles of adjuvant chemotherapy with carboplatin and paclitaxel, the patient developed large-volume ascites and peritoneal carcinomatosis. She showed a short-term response to weekly gemcitabine treatment and was started on pembrolizumab and lenvatinib five months after her initial diagnosis. A follow-up CT scan showed near-complete resolution of the ascites and omental caking. However, after receiving pembrolizumab and lenvatinib for 11 months, she was found to have a newly developed vaginal cuff mass and a newly enlarged right axillary lymph node on physical examination. Both were biopsied and confirmed to be metastatic carcinosarcomas. Subsequently, she experienced rapid disease progression and gradual deterioration of performance status. The patient died from acute hypoxic respiratory failure secondary to COVID-19, 21 months after her initial diagnosis (**Figure 3**).

Patient perspectives: not able to be obtained as the patient passed away.

### 2.4 Case 4

**Figure 4:**
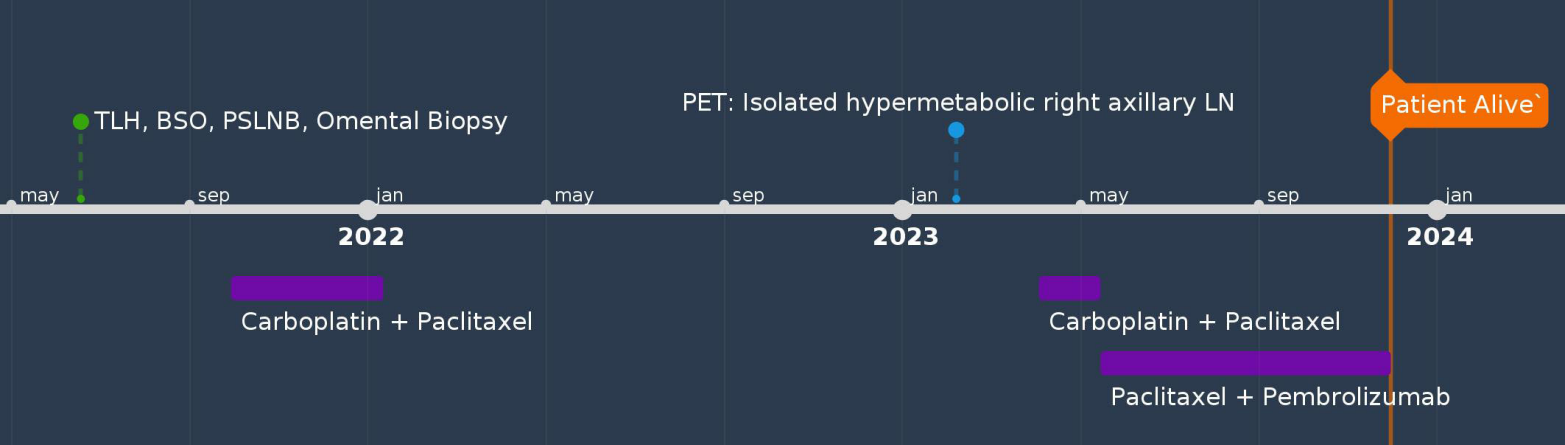
Timeline of Case #4. BPPLND, bilateral pelvic and paraaortic lymph node dissection; BSO, bilateral salpingo-oophorectomy; LN, lymph node; TAH, total abdominal hysterectomy; TLH, total laparoscopic hysterectomy; PLD, pegylated liposomal doxorubicin; PSLNB, pelvic sentinel lymph node biopsy; SBRT, stereotactic body radiation therapy.

A 63-year-old female (G3P3) with hypertension, rheumatoid arthritis, and hypercholesterolemia presented with PMB and abdominal cramps in 2021. Her brother had prostate cancer. Endometrial biopsy revealed uterine serous carcinoma. The patient underwent RATLH, BSO, pelvic sentinel lymph node biopsy, omental biopsy, and pelvic washing. The final pathology showed uterine serous carcinoma (tumor only on a polyp, no myometrial invasion, pT1a) with negative pelvic sentinel lymph node involvement (pN0), FIGO stage IA. Peritoneal fluid cytology was negative. IHC staining showed MMR proficiency, estrogen and progesterone receptor positivity, and p53 mutation. She was treated with four cycles of carboplatin and paclitaxel but declined vaginal brachytherapy. Twelve months after treatment, her CA-125 level had increased from the normal range to 127. CT scan of the abdomen and pelvis was unremarkable; however, a PET/CT revealed an isolated hypermetabolic pathologically enlarged right axillary lymph node. A biopsy of the axillary lymph node confirmed it to be a metastatic, poorly differentiated endometrial adenocarcinoma. She was started on carboplatin and paclitaxel treatment and had an allergic reaction to carboplatin in cycle two. Pembrolizumab was added to cycle 3 in combination with paclitaxel. Repeat CT scan showed that the right axillary lymph node had decreased in size, and the patient was recommended to undergo surgical axillary lymph node dissection followed by adjuvant radiation treatment and maintenance treatment with pembrolizumab. However, the patient declined surgery and remained on paclitaxel and pembrolizumab until this date, 29 months after her initial diagnosis (**Figure 4**).

Patient perspectives: The patient was pleased that the tumor responded to treatment and had decreased in size. However, she was concerned that surgical resection of the axillary mass would spread the tumor. The patient has been undergoing declining surgery.

## 3 Discussion

Axillary lymph node metastasis is uncommon in any type of gynecologic cancer and has not been well reviewed. In our series of four cases, three cases developed isolated axillary lymph node metastasis as the first site of recurrence. In one case, it was the harbinger of a second large-volume disease recurrence. Of the three patients with isolated axillary lymph node metastasis, two had long-term survival after aggressive locoregional treatment, including surgery and radiation. As of November 2023, three of the four patients were alive, under surveillance, or received systemic therapy. Their overall survival ranged from 24 months to 128 months after the initial diagnosis.

The majority of extrauterine spread in endometrial cancer involves the retroperitoneal lymph nodes. In a retrospective study from Italy on high-risk early-stage patients with endometrial cancer, the observed occurrence was 3.3% in both pelvic and para-aortic lymph nodes in those who underwent lymphadenectomy (7). In the evaluation of uncommon lymph node recurrence other than the above sites, an earlier single institutional study of 22 patients with endometrial cancer documented the first recurrence sites to be the inguinal, supraclavicular, and axillary lymph nodes (8). There were two cases of axillary lymph node metastasis: one had simultaneous supracervical lymph node metastasis, and the other had isolated axillary lymph node involvement. The latter patient had a history of stage III endometrial cancer at diagnosis and was treated with surgical excision and high-dose radiotherapy (≥5,000 cGy).

The metastatic route of gynecologic cancers to the axillary lymph node is postulated by two routes: (1) transthoracic lymphatic drainage or the transdiaphragmatic route, and (2) the posterior route (9). In the transdiaphragmatic route, such as in the presence of ascites and peritoneal carcinomatosis, tumor cells cross the diaphragm and invade the superior diaphragmatic lymph node. The tumor cells then drain toward the prepericardial and parasternal lymph nodes and continue directly to the internal jugular and subclavian veins or the subclavian lymph trunk and thoracic duct. The posterior route gathers all lymphatic pathways from the deep lymphatic vessels inferior to the diaphragm (iliac, para-aortic, and mesenteric) and superficial lymphatic vessels inferior to the level of the umbilicus. This lymphatic drainage unification forms the cisterna chyli and thoracic duct, which travels in the posterior mediastinum and drains into the junction of the left subclavian and internal jugular veins. Notably, drainage from the left subclavian and internal jugular veins to the axillary lymph nodes presumably follows the reverse route. In addition, isolated axillary lymph node recurrence also represents a “skipping” metastasis along the way. In a small series in which PET/CT was used to evaluate advanced-stage epithelial ovarian cancer, a significant number of patients had supradiaphragmatic lymph node metastasis (9).

When reviewing the routes of axillary lymph node metastasis, each case showed a unique pattern. Case #3 had widespread peritoneal carcinomatosis and retroperitoneal lymph node involvement, which likely contributed to right axillary lymph node metastasis through both the transdiaphragmatic and posterior routes. Case #1 had positive pelvic cytology and pelvic lymph node involvement, and both characteristics could potentially lead to disease spread via the transdiaphragmatic and posterior lymphatic drainage routes. However, in cases #2 and #4, neither the transdiaphragmatic nor the posterior route could explain the cause of the right axillary lymph node metastasis. There were no malignant cells in the pelvic washing or peritoneal carcinomatosis. Although case #2 was incompletely staged, the fact that she did not develop disease in the pelvic or para-aortic lymph nodes indicates that the posterior route of lymphatic drainage was not a contributing factor to axillary lymph node metastasis.

All four patients received adjuvant chemotherapy after their initial diagnosis because of aggressive histology. Patients in cases #1 and #2 received sequential chemotherapy and pelvic radiation based on multiple phase II study results available at that time (10, 11). Currently, systemic therapy is considered the foundation of adjuvant therapy in patients with extrauterine diseases. Two prospective randomized control studies (RCT) demonstrated the effectiveness of concurrent chemoradiation with cisplatin and external-beam radiation followed by carboplatin and paclitaxel in treating advanced-stage endometrial cancer (3, 12). For high-risk uterine cancer with disease limited to the uterus, we applied the treatment strategy endorsed by the GOG 249 study, which demonstrated the similar efficacy of either pelvic radiation or vaginal brachytherapy followed by carboplatin and paclitaxel (13) This treatment was used in case #4. Notably, concurrent chemoradiation with cisplatin and external-beam radiation followed by carboplatin and paclitaxel can be a reasonable option as well (3, 12) Patients in cases #1 and #3 also received pembrolizumab and lenvatinib at the time of disease progression and benefited from prolonged disease control (14, 15). This real-world evidence supports the efficacy of this regimen in both slow-growing, low-volume disease as well as rapidly progressing, high-volume disease of carcinosarcoma. With the newest evidence demonstrating survival benefits in treating advanced stage endometrial cancer, including carcinosarcoma, with immunotherapy in addition to front-line chemotherapy (16, 17), we expect to see a change in the treatment paradigm that may further impact the recurrence pattern.

All four patients had aggressive histology (serous carcinoma and carcinosarcoma) and either stage I or stage III disease at presentation. IHC and NGS testing showed no definite differences in predicting axillary recurrence (**Table 1**). However, isolated axillary lymph node metastasis, as presented in cases #1 and #2, appears to predict a disease characteristic of truly isolated oligometastasis and a more indolent course. Neither case #1 nor case #2 ever had axillary recurrence after receiving aggressive locoregional treatment for axillary lymph node metastasis with curative intent. These included axillary LND, radiation followed by systemic chemotherapy, and concurrent chemoradiation. Most importantly, neither case #1 nor case #2 developed widespread distant metastasis. The clinical progression pattern observed in cases #1 and #2 suggests that aggressive locoregional treatment could improve disease control in isolated axillary lymph node metastasis. Our observation is consistent with a previous study by Foote et al. that in their series of 22 patients with isolated peripheral lymph node recurrence, six patients remained free of disease at a median follow-up of 27 months. This observation and hypothesis were the basis for the recommendation of the treatment plan for case #4.

**Table 1:** Diagnosis, tumor characteristics, treatments, and outcome for the four cases.

	Case 1	Case 2	Case 3	Case 4
Pathology	Uterine serous carcinoma	Uterine carcinosarcoma	Uterine carcinosarcoma	Uterine serous carcinoma
Biomarkers	Positive: PAX-8, CK7, ER, PR, CA 125 Focally positive: WT1, BRST-2 Negative: TTF1, HER-2, CK20, mammaglobin	Positive: p53, PR Negative: SMM-1, p63, vimentin, ER, GCDFP-15, mammaglobin, CD10	Positive: PAX8, p53, GATA-3 Negative: ER, CK7, CK20	Positive: PAX-8, ER, PR, Negative: HER2
NGS markers	MSS, TMB-low; PPP2R1A, P179R, TP53, R248Q mutation	Not applicable	MSS, TMB-low; KRAS, BRD4, and TP53 mutation	MSS, TMB-low
Initial staging	IIIC1	Clinically stage I	IIIC2	IA
Time to first recurrence	29 months	25 months	3 months (disease progression while on chemotherapy)	12 months
Chemo after recurrence	Carboplatin and liposomal doxorubicin	Cisplatin (radiosensitizer)	Weekly Gemcitabine, followed by Pembrolizumab and Lenvatinib	Pembrolizumab, Carboplatin and Paclitaxel
Surgery, number of axillary LN removed	Left axillary lymphadenectomy, 19	Right axillary lymphadenectomy, 8	Not applicable	Not applicable
Adjuvant radiation	5,040 cGy to left axillary area	Concurrent chemoradiation 4,500 cGy to right axillary area	Not applicable	Not applicable
Second site of recurrence	Vagina and right inguinal lymph node	Not applicable	Vagina and right axillary lymph node	Not applicable
Time to second recurrence	6 months	Not applicable	11 months	Not applicable
Overall follow up duration	117 months	134 months	21 months	29 months
Current status	Alive, receiving immunotherapy	Alive, on surveillance, disease free	Deceased	Alive, receiving immunotherapy and chemotherapy

Of note, in cases #1 and #2, adjuvant radiation was applied to the axilla to ensure sterilization of cancer cells in all the potential tumor-involved lymph nodes, and no further axillary recurrence developed. A new method for intraoperative lymph node assessment was developed using optical coherence tomography (18). Lymph node dissection can become more accurate with the advancement of this technology,.

In conclusion, axillary lymph node metastasis as the first site of recurrence is uncommon and is more often seen in endometrial cancer with high-risk histologies. There was no direct association between isolated axillary lymph node metastasis and disease stage at diagnosis. This recurrence pattern may not be due to a disseminated recurrence or metastasis. We recommend aggressive loco-regional treatment in addition to systemic treatment to maximize the chance of long-term disease control.

## Data availability statement

The original contributions presented in the study are included in the article/supplementary material. Further inquiries can be directed to the corresponding author.

## Ethics statement

The studies involving humans were approved by the Maimonides Medical Center Institutional Review Board. The studies were conducted in accordance with the local legislation and institutional requirements. Written informed consent for participation was not required from the participants or the participants’ legal guardians/next of kin in accordance with national legislation and institutional requirements. Written informed consent was obtained from the participant/patient(s) for the publication of this case report.

## Author contributions

YAC: Writing – original draft, Writing – review & editing. MO: Writing – original draft. YX: Writing – original draft, Writing – review & editing.
